# Improving Information Distribution and Education Within Memory Clinic

**DOI:** 10.1192/bjo.2024.337

**Published:** 2024-08-01

**Authors:** Rashed Alhadi, Isabella Harley, Cara McDonald

**Affiliations:** NHS Tayside, Dundee, United Kingdom

## Abstract

**Aims:**

The aims of this project were to improve patient education and overall information distribution within the Memory Clinic within the Old Age Psychiatry department, based at Kingsway Care Centre, Dundee.

**Methods:**

This project originated, after there were concerns raised from relatives of a patient who had recently been assessed in the Memory Clinic. A suggested area for improvement included distributing information to patients, highlighting any potential tests or topics of conversation that may be explored during a Memory Clinic appointment. In response, our team engaged in a thorough collaboration with our colleagues in Psychiatry and the Post-Diagnostic Services (PDS). As a result of this partnership, a summary sheet was compiled, highlighting the spectrum of cognitive testing and assessments that may be conducted, potential medicinal treatments and other significant considerations, including driving and Power of Attorney statuses. To ensure these resources were both accessible and informative, they were systemically distributed to patients. The materials were paired with feedback forms to capture patient experiences and insights, to be later collected by the PDS.

**Results:**

Whilst this project remains in the data gathering stages, provisional data has been very promising in showing improvement in clarity of information delivered to patients (both in current and future assessments), explanation to patients regarding medication and treatment options, and overall patient satisfaction.

**Conclusion:**

Optimising educational resources for both patients and families attending the Memory Clinic through summary documentation can be utilised to improve overall patient satisfaction. Aiding patients’ understanding of their diagnosis and further management of this, allows them and their families to feel more included in their care and optimises the delivery of holistic care within Psychiatry of Old Age.

